# Meta-analysis of randomised trials compares mortality after transcatheter versus surgical aortic valve replacement

**DOI:** 10.1007/s12471-020-01418-w

**Published:** 2020-04-17

**Authors:** J. Vendrik, J. Baan Jr.

**Affiliations:** Heart Centre, Amsterdam University Medical Centres (location AMC), Amsterdam, The Netherlands

In this issue of the *Netherlands Heart Journal*, Takagi et al. [1] report a systematic review and meta-analysis regarding the treatment of patients with severe symptomatic aortic stenosis, with either a transcatheter (transcatheter aortic valve implantation, TAVI) or fully surgical (surgical aortic valve replacement, SAVR) approach. Their study provides us with a very thorough update on mortality after both procedures, using data gathered from all of the pivotal randomised trials [2–8]. Although none of the original analyses and current meta-analyses from the individual trials reported significantly lower mortality after TAVI than after SAVR, their pooled analyses of 7631 patients, including the most recent low-risk trials [3, 5], did show a significantly lower mortality associated with TAVI. The absolute risk reduction with TAVI is small, 0.6% and 1.1% for 30-day and 1‑year mortality, respectively. However, when combined with the fact that absolute mortality rates are already very low in current day practice, in addition to the ongoing increase in the number of TAVI procedures performed in the Netherlands [9], even these relatively small reductions may be of clinical significance. Hence, this article supports the ongoing broadening indication for TAVI and the gradual shift toward TAVI becoming the preferred treatment strategy in the majority of patients with severe symptomatic aortic stenosis. However, a few caveats in the current literature and in our knowledge still remain, the most prominent being long-term valve durability. Since the studies included in this systematic review, as the authors properly acknowledge, do not report on long-term follow-up, no conclusions can be drawn regarding long-term valve durability. All known data in high-risk and inoperable patients show acceptable and, more importantly, similar or lower rates of structural valve deterioration (SVD) in TAVI-treated patients than in SAVR-treated patients [10]. To date, results of long-term follow-up in low-risk patients are available only from the NOTION trial [11], showing a lower 6‑year rate of SVD in transcatheter valves than in surgical aortic bioprostheses (4.8% vs 24%; *p* < 0.001). However, in this trial earlier-generation prostheses (both transcatheter and surgical) were implanted, and newer prostheses may yield different, better long-term results. In vitro testing of the latest SAPIEN 3 aortic prosthesis showed excellent results up to the equivalent of 25 years in nominally expanded valves, comparable with the newest surgically implanted prostheses [12]. Since the PARTNER 3 [3] and Evolut R Low Risk [5] trials will provide us with much awaited long-term echocardiographic data on low-risk patients treated with the newest prostheses, patience is required in this regard.

This, however, raises the next caveat in our knowledge. In the most recent low-risk trials, patients were treated only via a transfemoral approach (100% for the PARTNER‑3 [3] and 99% for the Evolut Low Risk RCT [5] respectively). Hence no conclusions can be drawn regarding TAVI using different access routes. As a large proportion of the screened patients (302/1435) in the PARTNER 3 trial were not included due to anatomical exclusion criteria, the subgroup of patients who cannot undergo transfemoral (TF-) TAVI can be substantial [3]. Since alternative access routes are per definition more invasive than TF-TAVI, and often reflect a worse preoperative patient health status, extrapolation of these data to other subgroups of patients, and comparing these to those of surgically treated patients, can only be done with extreme caution.

Thirdly, one of the most prominent TAVI-related complications is the need for permanent pacemaker implantation (NPPMI). Takagi et al. describe a risk difference of +8.89% for NPPMI at 30 days for the TAVI-treated patients. Pacemaker implantation does not influence short- and mid-term mortality [1, 13, 14], but may negatively influence long-term mortality in theory, especially in completely pacemaker-dependent patients. The need for NPPMI is highly dependent on the valve system used. As reported in both the simultaneously published low-risk trials, which showed 17.4% [5] and 6.6% [3] for the TAVI patients in the Evolut Low-Risk and PARTNER 3 trials, respectively, as well as in large, pooled analyses [15], NPPMI rates are substantially higher when self-expandable valves are used. In the PARTNER 3 trial, the NPPMI rate was not significantly higher in the TAVI-treated than in the SAVR-treated patients (6.6% vs 4.1%). As younger and healthier, lower-risk patients are treated, with fewer risk factors for NPPMI [16], and as implantation techniques evolve [17, 18] and algorithms are created, avoiding futile pacemaker implantation [19], NPPMI rates may decrease further until they reach the SAVR range.

Lastly, although post-procedural mortality is the most important and hard endpoint, it is not the only one. Especially for the population of fragile, elderly patients, softer endpoints such as a short period of hospitalisation, quick recovery, symptomatic improvement and quality of life may be just as important. In the PARTNER 3 data, the median length of hospitalisation was 3 days after TAVI, and 7 days after SAVR. Furthermore, a significantly larger proportion of the TAVI-treated patients were discharged to their own home (95.8% vs 73.1%). Several early-discharge protocols have been published (FAST-TAVI [20], 3M-TAVR [21]) to further facilitate short hospital stays and possibly quicker recovery [22, 23]. In this regard, the PARTNER 3 data show us that 30 days after the procedure only 19.7% of the TAVI-treated patients had dyspnoea (New York Heart Association class ≥2, versus 33.3% in the SAVR group), whereas TAVI-treated patients walked 32% further during the 6‑min walk test and scored 38% better on the Kansas City Cardiomyopathy Questionnaire score. All these outcomes are similar for both approaches at 1‑year follow-up, depicting a quicker recovery for TAVI-treated patients. Although all these findings need to be further confirmed with real-life data, they do support the evidence that the treatment paradigm is justly shifting towards TAVI.

In conclusion, Takagi et al. provide us with a much appreciated systematic review, guiding current treatment of patients with aortic valve stenosis. Several challenges need to be overcome in the future. However, current data reflect significant benefits for TAVI over SAVR in the majority of patients with severe symptomatic aortic valve stenosis.
